# Direct costs of treating men with prostate cancer with *High Intensity Focused Ultrasound* ^*^

**DOI:** 10.1590/1980-220X-REEUSP-2023-0132en

**Published:** 2023-11-27

**Authors:** Pâmela Adalgisa Lopes da Silva, Antônio Fernandes Costa Lima

**Affiliations:** 1 Universidade de São Paulo, Escola de Enfermagem, Programa de Pós-Graduação em Gerenciamento em Enfermagem, São Paulo, SP, Brazil.; 2 Universidade de São Paulo, Escola de Enfermagem, Departamento de Orientação Profissional, São Paulo, SP, Brazil.

**Keywords:** Prostatic Neoplasms, Therapeutic Uses, Ultrasound, High-Intensity Focused, Transrectal; Hospital Care, Costs and Cost Analysis, Direct Service Costs, Neoplasias de la Próstata, Usos Terapéuticos, Ultrasonido Enfocado Transrectal de Alta Intensidad, Atención Hospitalaria, Costos y Análisis de Costo, Costos Directos de Servicios, Neoplasias da Próstata, Usos Terapêuticos, Ultrassom Focalizado Transretal de Alta Intensidade, Assistência Hospitalar, Custos e Análise de Custo, Custos Diretos de Serviços

## Abstract

**Objective:**

To analyze the direct costs of materials, medicines/solutions and healthcare professionals required to treat men with prostate cancer using *High Intensity Focused Ultrasound*.

**Method:**

Quantitative, exploratory-descriptive research, single case study type. Data were collected from electronic medical records/printed documentation from the Operating Room of a public teaching and research hospital. Health professionals estimated the respective time spent on activities in the following stages: “Before anesthetic induction”, “Before performing thermal ablation”, “During thermal ablation” and “After performing thermal ablation”. Costs were calculated by multiplying the (estimated) time spent by the unit cost of direct labor, adding to the measured cost of materials, medicines/solutions.

**Results:**

The measured costs with materials corresponded to US$851.58 (SD = 2.17), with medicines/solutions to US$72.13 (SD = 25.84), and estimated personnel costs to US$196.03, totaling US$1119.74/procedure.

**Conclusion:**

The economic results obtained may support hospital managers in the decision-making process regarding the adoption of the *High Intensity Focused Ultrasound* for the treatment of prostate cancer.

## RESUMO

**Objetivo:**

Analisar os custos diretos com materiais, medicamentos/soluções e profissionais de saúde requeridos à realização do tratamento de homens com câncer de próstata via *High Intensity Focused Ultrasound*.

**Método:**

Pesquisa quantitativa, exploratória-descritiva, do tipo estudo de caso único. Coletaram-se os dados em prontuários eletrônicos/documentações impressas do Centro Cirúrgico de um hospital público de ensino e pesquisa. Profissionais de saúde estimaram os respectivos tempos despendidos em atividades constantes das etapas: “Antes da indução anestésica”, “Antes da execução da termoablação”, “Durante a execução da termoablação” e “Após a execução da termoablação”. Calcularam-se os custos multiplicando-se o tempo (estimado) despendido pelo custo unitário da mão de obra direta, somando-se ao custo mensurado dos materiais, medicamentos/soluções.

**Resultados:**

Os custos mensurados com materiais corresponderam a US$851,58 (DP = 2,17), com medicamentos/soluções a US$72,13 (DP = 25,84) e os custos estimados com pessoal a US$196,03, totalizando US$1119,74/procedimento.

**Conclusão:**

Os resultados econômicos obtidos poderão subsidiar os gestores hospitalares no processo decisório quanto à adoção do *High Intensity Focused Ultrasound* para o tratamento do câncer de próstata.

## RESUMEN

**Objetivo:**

Analizar los costos directos de materiales, medicamentos/soluciones y profesionales de la salud necesarios para tratar a hombres con cáncer de próstata a través de *High Intensity Focused Ultrasound*.

**Método:**

Investigación cuantitativa, exploratoria-descriptiva, tipo estudio de caso único. Los datos se obtuvieron de registros médicos electrónicos/documentación impresa del Centro Quirúrgico de un hospital público de enseñanza e investigación. Los profesionales de la salud estimaron el tiempo respectivo dedicado a las actividades en las siguientes etapas: “Antes de la inducción anestésica”, “Antes de realizar la ablación térmica”, “Durante la realización de la ablación térmica” y “Después de realizar la ablación térmica”. Los costos se calcularon multiplicando el tiempo (estimado) invertido por el costo unitario de la mano de obra directa, sumándolo al costo medido de materiales, medicamentos/soluciones.

**Resultados:**

Los costos medidos con materiales correspondieron a US$851,58 (DE = 2,17), con medicamentos/soluciones a US$72,13 (DE = 25,84) y los costos de personal estimados a US$196,03, totalizando US$1119,74/procedimiento.

**Conclusión:**

Los resultados económicos obtenidos podrán apoyar a los gestores hospitalarios en el proceso de toma de decisiones respecto a la adopción del *High Intensity Focused Ultrasound* para el tratamiento del cáncer de próstata.

## INTRODUCTION

Prostate cancer (PCa) consists of a process in which a sequence of genetic changes transform normal cells into malignant cells, usually presenting silent progression in its initial phase^(1)^. However, in advanced stages, there is the presence of pain when urinating, difficulty urinating, such as frequent urination, dysuria, and nocturia, and infection processes^(2)^.

Treatments aimed at men with PCa are directed to preventing death and disability, minimizing complications related to interventions. The most frequent ones are radical prostatectomy (RP), external beam radiotherapy, brachytherapy, cryotherapy, and androgen deprivation therapy^(3)^, determined through stratification of the risk of PCa recurrence and progression^(4)^.

Patients with very low risk stratification tumors, with stage T1c, Gleason score ≤ 6, prostate specific antigen (PSA) < 10 ng/ml, less than three positive biopsy fragments with ≤ 50% involvement in each fragment and PSA density < 0.15 ng/ml/g may undergo active surveillance, external beam radiotherapy, brachytherapy, and radical prostatectomy (RP). In cases of low-risk tumors, with stage ≤ T2a, Gleason score ≤ 6, PSA < 10 ng/ml, and intermediate risk, with stage T2b or T2c, Gleason Score 7 and PSA 10–20 ng/ml, active surveillance, RP, external radiotherapy, and brachytherapy are indicated. Patients with high-risk tumors, stage ≥ T3, Gleason score ≥ 8, PSA > 20 ng/ml can be treated with external radiotherapy associated with long-term hormone therapy (HT) and RP, extended pelvic lymphadenectomy^(4)^.

Among the therapeutic options, international literature has highlighted the increasing use, over the years, of *High Intensity Focused Ultrasound* (HIFU), ultrasonic energy for the primary treatment of PCa or rescue treatment after previous radiotherapy. It is indicated for men with multiple comorbidities, with PCa in stage T1 or T2 and with a Gleason Score less than or equal to seven, PSA level lower than 15 ng/ml and prostate volume lower than 40 ml^(5)^. It is a hospital procedure, without incision, free of radiation, used to destroy the structure or part affected by PCa, through thermoablation, preserving neighboring organs, urinary sphincter, and erect nerves, minimizing side effects such as erectile dysfunction and urinary incontinence^(6)^.

In Brazil, despite the use of robotic high-intensity ultrasound therapy equipment being approved by the Brazilian Health Regulatory Agency (ANVISA)^(7)^, the HIFU procedure is not included in the Table of Procedures, Medications, Orthoses, Prostheses, and Special Materials of the Brazilian Public Health System (*SUS*)^(8)^ and, therefore, it is not a therapy yet available in the public health network.

HIFU, aimed at treating men with PCa, is available in six Brazilian institutions, which serve the Supplementary Health System, and in two reference centers for the training of urologists linked to the *SUS*. This therapeutic procedure has been performed in the country for more than ten years, as well as in the largest international centers in Europe, North America, Asia, and Oceania. However, in most countries that carry it out, it is approved by regulatory agencies, with reimbursement occurring through various paying sources^(9)^.

In the national territory, according to opinion no. 15/2020 of the Federal Medical Council, the HIFU procedure will continue to be carried out in clinical research environments, until its therapeutic role (efficacy and safety) for the treatment of patients with localized PCa is proven, complying with the protocols established and authorized in the Research Ethics Committees/National Research Ethics Committee System, through future studies. The aforementioned opinion explained that, at the time of its issuance, a randomized multicenter study, called Chronos study, was underway and aimed at comparing radical surgeries and HIFU, observing free time or the absence of disease recurrence. It is believed that such a study could be a predictor in the treatment of PCa with HIFU in the national context^(10)^.

In 2017, a large Brazilian public teaching and research hospital (HPEP), a reference in men’s health, set up and implemented HIFU for the treatment of PCa via *SUS*. Accordingly, it acquired high-intensity ultrasound equipment (Focal One) and imported supplies, adapted the structure of a room in the Operating Room, and provided specific training programs for a fixed group of urologists and anesthetists, nurses and nursing technicians to participate in this procedure.

Considering the indispensability of knowing the financial repercussions that investments in health technology generate for institutions, given that the provision of services in the health sector is costly, this study aimed to analyze the direct costs of materials, medicines/solutions, and health care professionals required to treat men with PCa through HIFU.

## METHOD

### Design of Study

This is a quantitative, exploratory/descriptive study, in the form of a single case study.

### Local

The research was conducted in the Operating Room of an HPEP, a pioneer in the setting and implementation of HIFU in the *SUS* and chosen for having an adequate technological structure and quantitative and qualitative human resources required to meet the care demands of men with PCa.

A fixed group of professionals works in the HIFU procedure: the urology medical team, which specialized in France and uses the same surgical technique; residents in urology; anesthetists, nurses, and nursing technicians, duly trained to carry out activities related to the procedure.

### Population and Selection Criteria

It consisted of electronic and printed documentation, relating to the performance of 192 HIFU procedures, between April 2017 and August 2020, in 192 patients with PCa between stages T1 and T2, with a Gleason score ≤ 7, post-radiotherapy procedure, and patients who previously received HIFU therapy.

It should be clarified that from March to October 2019 high-intensity ultrasound equipment (*Focal One*) presented technical failures, and HIFU at HPEP was interrupted. Due to the Covid-19 pandemic, this procedure began to be scheduled sporadically and was temporarily suspended from August 2020 on. Subsequently, given the magnitude of the pandemic situation, the suspension was maintained for an indefinite period, notably due to the impossibility of HPEP purchasing some imported inputs, due to the high costs required to carry out HIFU.

The variables of this study consisted of surveying the quantity and type of materials, medicines/solutions consumed, and estimating the time spent by health professionals in the four sequential stages required to allow the performance of HIFU: 1) “Before anesthetic induction”, 2) “Before performing thermal ablation”, 3) “During thermal ablation”, and 4) “After performing thermal ablation”.

### Data Collection

From April to August 2022, data regarding the consumption of materials and medicines/solutions used in HIFU procedures were collected. Subsequently, a form, built specifically for the study and previously tested, was applied to health professionals, according to their professional category, to determine, individually, the estimated time spent to carry out the respective activities included in the HIFU procedure, distributed in the four stages mentioned above. Considering that the estimate would be based on the accumulated expertise, through the performance of this procedure over two years, urology residents were not included in this study.

The times estimated by professionals who worked together in some activities were added and divided by the number of respondents, obtaining the average time. As it is a standardized procedure and carried out by the same health professionals, no significant differences were found in time estimates.

To estimate average direct costs (ADC) for healthcare professionals, direct labor costs (DLC) were calculated from average salaries provided by the HPEP’s Human Resources Department.

DLC concerns personnel who work directly to obtain a product or service, with the possibility of identifying the time spent and the person performing the work. It consists of salaries, social security contributions, vacation provisions, and 13th month pay^(11)^.

The calculation of the weighted average salary for the anesthetist category corresponded to US$4,330. 10/month (80 contractual hours), obtaining an average cost/hour of US$54.12 and a minute of US$0.90; in the urologist category, to US$2,198.51/month (80 contractual hours), US$27.48/hour, and US$0.46/minute; in the nurse category, to US$1,332.73/month (180 contractual hours), US$7.41/hour, and US$0.12/minute; and the nursing technician category, to US$788.27/month (180 contractual hours), US$4.38/hour, and US$0.07/minute.

To measure the ADC of supplies, those responsible for the Purchasing Department/Warehouse were asked for information regarding the costs of the latest acquisitions of materials and solutions/medicines.

The costs with supplies and DLC with health professionals were converted to the US dollar – US$, based on the quote provided by the Brazilian Central Bank, on 11/18/2021 (R$5.5464/US$1.00).

The total ADC was obtained by multiplying the (estimated) time spent by the unit cost of the DLC, adding to the measured cost of materials, solutions and medicines^(12)^.

### Data Analysis and Treatment

Data were organized in an electronic spreadsheet, through independent double typing and, subsequently, treated using descriptive and inferential statistics.

### Ethical Aspects

The study fully complied with all the specifications of resolution no. 466, of December 12, 2012, which approves the guidelines and regulatory standards for research involving human beings. The Research Ethics Committees of the proposing institution and HPEP (co-participating institution) approved the study in 2021, through consubstantiated opinions, numbers 4.655.519 and 4.908.373, respectively. All health professionals performing the HIFU procedure agreed to participate in the research, by signing the Informed Consent Form.

## RESULTS

When performing the 192 HIFU procedures, the measured ADC related to material consumption ranged from US$846.67 to US$859.38, with an average of US$851.58 (SD = 2.17); the items with the highest unit cost were *Focal Pack Kit* (US$829.32) and the surgical warming blanket (US$14.33). A total of 8,218 materials were consumed, corresponding to 50 different items, totaling US$163,506.95 (100.00%), with US$159,229.44 (97.38%) referring to 192 *Focal Pack Kits* and US$2,751.36 (1.68%) for 192 surgical warming blankets.

The *Focal Pack Kit* consists of a transfer bag with bubble retention system, an Ablasonic® bag with 350 ml, a 50 ml syringe with *luer-lock* connection, a perforator, two tubes, a latex balloon, a reflector for an infrared sensor, and a tube with 120 ml acoustic gel, all disposable.

The measured ADC of medicines/solutions ranged from US$17.45 to US$102.28, with an average of US$72.13 (SD = 25.84); the drugs with the highest unit cost were the anesthetic Sevoflurane (US$59.13), the opioid remifentanil hydrochloride (US$7.21 unit), and the neuromuscular blocker Cisatracurium Besylate (US$5.59). A total of 4,952 medicines/solutions were consumed, related to 39 items, totaling US$13397.48 (100.00%), with the drugs Sevoflurane (US$8455.59 – 63.11%), Cisatracurium Besylate (US$1145, 95 – 8.55%), and Cefuroxime sodium 750 mg (US$1128.96 – 8.43%) being the items that most contributed to these costs.

With regard to the characterization of the 10 health professionals who estimated the time spent carrying out the respective activities comprising the four stages of the HIFU procedure, it can be seen, in Table 1, that two were nurses, four were nursing technicians, two were urologists, and two were anesthetists. These professionals’ average age corresponded to 35.40 years (SD = 8.60), the average time of training to 11.90 years (SD = 6.60), and the average time of work in the Operating Room to 10.20 years (SD = 6.60).

**Table 1:** Distribution of professionals performing the HIFU procedure according to professional category, quantity, average age, average time since graduation, and average time working in the HPEP Operating Room – São Paulo, SP, Brazil, 2022.

Professional category	N	%	Average age (SD^*^)	Average time since course completion	Average time working in the Operating Room
Nurse	2	20.00	33.00(SD = 11.30)	9.00(SD = 5.60)	7.00(SD = 2.80)
Nursing technician	4	40.00	34.50(SD = 10.30)	10.50(SD = 8.30)	10.50(SD = 8.90)
Anesthetist	2	20.00	44.00(SD = 4.24)	18.50(SD = 3.50)	17.00(SD = 5.60)
Urologist	2	20.00	31.00(SD = 0.00)	5.00(SD = 0.70)	4.50(SD = 0.00)
**TOTAL**	**10**	**100.00**	**35.40(SD = 8.60)**	**11.90(SD = 6.60)**	**10.20(SD = 6.60)**

^*^SD – standard deviation.

Among the nursing professionals, 83.30% were women, with an average age of 34 years (SD = 9.5), average time since course completion of 11.2 years, and average time working in the HPEP Operating Room of 9.7 years. Among the nursing professionals, 75.00% were men, with an average age of 37.50 years (SD = 7.9), average time since course completion of 13 years, and average time working in the HPEP Operating Room of 11 years.

It is shown, in Chart 1, that the estimates of the time spent and the total ADC with the DLC of health professionals, in the four sequential stages comprising the HIFU procedure, corresponded to 384 minutes and US$196.03 (100.00%), respectively. The second (“Before performing the thermal ablation with HIFU”) and the third (“During thermal ablation with HIFU”) stages were the most significant for the composition of these estimates: 64 minutes (16.67%) and US $46.03 (23.48%), and 213 minutes (55.47%) and $101.13 (51.59%), in that order.

**Chart 1:** Distribution of the four stages of the HIFU procedure, according to the estimated time spent and the ADC with the DLC of the healthcare professionals performing it – São Paulo, SP, Brazil, 2022.

Stages	Estimated time (minutes) – %	Estimated ADC* (US$^†^) – %
First: “Before anesthetic induction”	49 – 12.76	17.47 – 8.91
Second: “Before performing thermal ablation with HIFU”	64 – 16.67	46.03 – 23.48
Third: “Before thermal ablation with HIFU”	213 – 55.47	101.13 – 51.59
Fourth: “After thermal ablation with HIFU”	58 – 15.10	31.40 – 16.02
**TOTAL**	**384 – 100.00**	**196.03 – 100.00**

* ADC – Average direct cost, ^†^US$ – US dollar with conversion rate: R$5.54/ US$1.00. Brazilian Central Bank, based on the quote on 11/18/2021.

In the first stage, “Before anesthetic induction”, started in the post-anesthesia care unit (PACU), with an ADC of US$17.47 (100.00%), the activity “Assembly and preparation of the HIFU device”, in charge of the urologist, presented the highest estimate of time spent (15 minutes), as well as the highest estimated ADC (US$6.75–38.63%). The activities “Embracement in the operating room and provision of guidance regarding the procedure/clarification of doubts” and “Transferring the client from the PACU to the operating room and positioning on the operating table” also had the highest estimated time expenditure (11 and 10 minutes, respectively). However, “Peripheral vascular access puncture”, performed by the anesthetist, was the second activity with the highest estimated ADC (US$4.50–25.75%).

Regarding the second stage, “Before performing thermal ablation through HIFU”, ADC of US$46.03 (100.00%), the activity with the highest estimated time and ADC was “Positioning the client on the surgical table and placing cushions”, carried out jointly by the nursing technician, anesthetist, and urologist, corresponding to 15 minutes and US$20.85 (42.29%). Two other activities performed by the anesthetist (“preparation and administration of anesthesia” and “orotracheal intubation”) then had the highest estimated time and ADC, 13 minutes – US$11.70 (25.42%) and 11 minutes – US$9.00 (19.55%), respectively.

As for the third stage, “During the execution of thermal ablation through HIFU”, with an ADC of US$101.13 (100.00%), the activities with the highest estimated time and ADC were “Maintenance of anesthesia”, 66 minutes – US$59.40 (58.73%) and “Performance of thermal ablation”, 66 ­minutes – US$30.36 (30.02%), performed by the anesthesiologist and urologist, sequentially. Despite the estimated time for the Nursing Technician work in the activity of “Circulation in the room” (66 minutes), the ADC (US$4.62–4.56%) did not have a significant impact on the composition of the costs of this stage.

With regard to the fourth stage, “After performing thermal ablation with HIFU”, the estimated ADC was US$31.40 (100.00%), with “Orotracheal extubation”, performed by the anesthetist, being the activity with the highest estimate of time and ADC, corresponding to 15 minutes and US$13.50 (42.99%). Another activity performed by the anesthetist (“Removal of drapes, cushions, positioning of the client on the surgical table, and transfer to the transport stretcher”) had the second highest estimated ADC (US$6.30–20.06%).

Adding the ADC measured with materials, medicines/solutions and the ADC estimated with the DLC of health professionals, we obtained a total ADC of US$ 1119.74 (100.00%) per HIFU session, with the predominance of representativeness of ADC measured with materials, US$851.58 (76.05%), as indicated in Chart 2 below.

**Chart 2:** Distribution of the ADC of the HIFU procedure, according to the costs calculated with materials and medicines/solutions, estimated costs with DLC of healthcare professionals and total ADC per HIFU session – São Paulo, SP, Brazil, 2022.

Variables	ADC* (US$^†^)
ADC* calculated with materials	851.58 – 76.05
ADC* determined with medicines/solutions	72.13 – 6.44
ADC* estimated with DLC of healthcare professionals	196.03 – 17.51
**TOTAL ADC** * **– US$** ^†^	1119.74 – 100.00

* ADC – Average direct cost, ^†^US$ – US dollar with conversion rate: R$5.54/ US$1.00. Brazilian Central Bank, based on the quote on 11/18/2021.

## DISCUSSION

Faced with the increasing emergence of new cases of cancer, studies have proven, worldwide, the indispensability of greater financial incentives for research, making health innovation necessary in the treatments provided to the population. In Brazil, the financial impact of treating cancer patients in the SUS is a reflection of dependence on the international market. To minimize this, the relevance of increasing the public budget in the area of research and innovation of oncological goods and services is highlighted, as already carried out in Europe, Asia, and the United States of America (USA)^(13)^.

Admittedly, technological-scientific advances present great growth and contributions to patients’ health, but, on the other hand, they require high investments and generate high operational costs^(14)^. From this perspective, it should be noted that Technological Assessment in Health is a strategy that contributes to the support for decision-making regarding the implementation of new technology^(15)^.

In hospital organizations, controlling and reducing costs, ensuring the quality of health services provided to the population, are challenges for managers, with knowledge of work processes being extremely important to increase their economic performance^(16,17)^. In this context, nursing professionals, in view of their healthcare role, need to know the costs of material resources, avoiding/minimizing waste, qualifying the decision-making process regarding adequate allocation and rational use, increasing the efficiency of processes, and contributing to the financial sustainability of healthcare organizations^(18)^.

In the financial aspect, the novelty of this absorption micro-costing study of the HIFU procedure is evident, which gives visibility to the measured costs of the consumption of materials, medicines/solutions and to the estimated costs with DLC of the required health professionals.

In fact, there were significant costs associated with some items of materials consumed during the performance of the procedures under study, notably the predominance of costs with the 192 Focal Pack Kits. It should be clarified that the Focal Pack Kit covers a set of items that help reduce the temperature of the probe, protecting the structures that surround the treatment region, such as the rectum, and the gel included in the kit helps in the propagation of ultrasound waves, generating better image quality^(19)^. However, the rational use of items with the highest unit cost was observed, with no occurrence of waste, especially as it is a properly standardized procedure carried out by the same health professionals.

In this study, all data relating to the quantity of materials, medications and solutions used in the HIFU procedure were extracted from the records of urologists and anesthetists, nurses and nursing technicians, contained in the electronic and printed medical records used in the HPEP Operating Room, which were adequate and complete.

It is worth noting that the patient’s medical record consists of an aggregate of ethical-legal documents belonging to the patient. However, the records made by professionals are the responsibility of both themselves and the institutions, as is the confidentiality of the data contained in the documents, and failure to adequately fill documentation relating to the assistance provided is considered a legal infraction^(20)^.

In the economic-financial dimension, hospital billing depends on records made by professionals and, for this reason, every medical record, in addition to being a form of communication within the health team professionals, is also a means of proving the care provided to the patient. Therefore, all records must be strictly correct, aiming to reduce financial losses, optimize deadlines, and improve work processes^(21)^.

Despite repeated searches of the national and international literature, no other absorption micro-costing studies were found that covered the variables addressed in this study. However, the predominance of costs associated with material resources was demonstrated, similar to recent studies addressing different procedures^(22–24)^.

A quantitative, exploratory-descriptive case study, which measured the ADC of 101 peripherally inserted central catheter (PICC) passages, by nurses, in a pediatric and neonatal intensive care unit, with 70 successful passages, found that, regardless of the outcome of the PICC passage, the cost of materials had a greater impact on this procedure ADC. Among the successful passages, the ADC with nursing professionals corresponded to US$ 6,409.15 (SD = 32.98) and, with materials, US$ 10,523.24 (SD = 75.11); among the unsuccessful passages, the ADC with nursing professionals was US$ 2,256.34 (SD ± 23.42) and with materials was US$ 3,496.88 (SD ± 54.34). The authors showed that the results obtained were in line with the findings of micro-­costing studies published nationally, in which the calculation of the ADC with materials was superior to the ADC with DLC of healthcare professionals^(22)^.

A quantitative, exploratory-descriptive study, in the form of a single case study, carried out in a public hospital in the State of São Paulo, found the ADC of long-term central venous catheter insertion in patients undergoing hemodialysis. A total ADC of US$134.56 (SD ± 3.65) was obtained, of which US$107.01 (DP ± 0.23) with material, US$22.10 (SD ± 3.63) with DLC from the catheter insertion, US$4.65 (SD ± 0.00) with medications, and US$0.80 (SD ± 0.15) with solutions. The high impact of material resource costs was noted and the need for their rational allocation was indicated, especially in public teaching and research hospitals, which have scarce and limited financial resources^(23)^.

Another quantitative study, also in the form of a single case study, observed the insertion of 139 PICCs in patients admitted to an adult cardiopulmonary intensive care unit. The total ADC for PICC insertion was US$286.04 (SD ± 39.49), with US$259.81 (SD ± 36.94) referring to the ADC with material and US$26.22 (SD ± 9 .01) to the nurses’ DLC ADC. It was concluded that knowledge about the time spent, quantity, and costs of resources involved in the procedure is essential, aiming to support assistance, educational, and managerial actions^(24)^.

In view of the above, it is believed that the economic scenario challenges, experienced in national and international contexts, require health professionals to master the management of material resources associated with knowledge of process components, interprofessional relationships, environmental, political, sociodemographic, and economical determinants. Such components are essential in the administration of institutions, as they allow institutional organization, favoring the development of care and management processes, providing improvements and added value^(25)^.

### Implications for Practice

This original study of absorption micro-costing of the HIFU procedure, aimed at treating PCa patients, gave visibility to the measured costs of the consumption of materials, medicines/solutions, and the estimated costs with DLC of the required healthcare professionals. It contributes to the verticalization of knowledge about the financial aspects associated with procedures carried out in the Operating Room, whose publications are still scarce, allowing cost management, aiming to control/minimize them, without compromising the quality of care.

### Limitation of the Study

The impossibility of carrying out non-participant observations of health professionals performing HIFU, due to the suspension, for an indefinite period, of the procedure at HPEP, because of the Covid-19 pandemic, may be indicated as a limitation of this study, which resulted in the lack of more in-depth process mapping.

## CONCLUSION

The measured ADCs with materials corresponded to US$851.58 (SD = 2.17), with medicines/solutions at US$72.13 (SD = 25.84) and estimated ADCs with personnel at US$196.03, totaling the total ADC of US$1119.74 per HIFU procedure.
